# Abrupt suspension of probiotics administration may increase host pathogen susceptibility by inducing gut dysbiosis

**DOI:** 10.1038/srep23214

**Published:** 2016-03-17

**Authors:** Zhi Liu, Wenshu Liu, Chao Ran, Jun Hu, Zhigang Zhou

**Affiliations:** 1 Key Laboratory for Feed Biotechnology of the Ministry of Agriculture, Feed Research Institute, Chinese Academy of Agricultural Sciences, No. 12 Zhongguancun South St., Beijing 100081,PR China

## Abstract

In this study, we investigated the risk associated with suspension of probiotics administration in tilapia, an animal model that may mimic immune-compromised conditions in humans. Tilapias were fed for 14 days using a probiotics-supplemented diet, followed by a three-day suspension of probiotics treatment and a subsequent challenge by *Aeromonas hydrophila*. Unexpectedly, the suspension of a probiotic strain *Lactobacillus plantarum* JCM1149 significantly triggered susceptibility of the host to *A. hydrophila*. We further observed that suspension of JCM1149 resulted in host gut microbiota dysbiosis and the subsequent disorder in the intestinal metabolites (bile acids, amino acids, and glucose) and damage in the intestinal epithelium, giving rise to a condition similar to antibiotics-induced gut dysbiosis, which collectively impaired tilapia’s gut health and resistance to pathogenic challenges. Additionally, we determined that JCM1149 adhered relatively poorly to tilapia intestinal mucosa and was rapidly released from the gastrointestinal tract (GIT) after suspension, with the rapid loss of probiotic strain probably being the direct cause of gut dysbiosis. Finally, three other probiotic *Lactobacillus* strains with low intestinal mucosa binding activity showed similar rapid loss phenotype following administration suspension, and induced higher host susceptibility to infection, indicating that the risk is a generic phenomenon in *Lactobacillus*.

A vast number of microbial cells, approximately ten times more than host cells, reside in vertebrate and mammal gastrointestinal tract (GIT), and have been proven to maintain and modulate the balance of gut environment^1^,^2^,^3^,^4^. Initiating at birth, gut microbiota reach an intestinal niche, gradually adapting to and decorating the host GIT environment. GIT homeostasis is one of critical performances of host gut health, playing a crucial role in protection against pathogenic infection^5^.

Gut microbiota directly combat the adhesion and proliferation of exogenous organisms by producing various broad-spectrum antimicrobial components, such as organic acids, diacetyl, and hydroperoxide. They also produce bactericidal proteins, and change local redox status, pH and nutrient distribution of certain GIT micro-environment^6^. The gut microbiota is also a key determinant in fortification of the host’s “GIT defense barrier”. Intestinal microbial populations metabolize otherwise indigestible complex carbohydrates and synthesize short chain fatty acids (SCFAs) such as acetate, propionate, and butyrate. SCFAs are the predominant energy source for epithelial cells, and benefit the refreshment and reorganization of the intestinal epithelia and their junctions^7^. Additionally, gut microorganisms directly regulate many important physiological pathways associated with intestinal cells, including pathways involved in the synthesis of proinflammatory cytokines and reactive oxygen species^8^. Intestinal microorganisms and their structural components, such as lipopolysaccharides, peptidoglycans, nucleotides, proteins, and lipoproteins, can be recognized by the pattern recognition receptors of host immune cells, mostly Toll like receptors (TLRs) and nucleotide binding oligomerization domain agents (NODs), resulting in stimulation of host immune responses^3^,^5^,^9^. Also, intestinal alkaline phosphatase (IAP)^10^, heat shock proteins^11^, amino acids^12^, bile acids^13^, hormones^14^, and vitamins^12^,^15^, mainly as GIT metabolites, are closely related with intestinal physiological function, and have been used as biomarkers of GIT homeostasis and health.

Once gut microbiome homeostasis disrupted, dysbiosis occurs, often leading to conditions that are favorable for pathogenic introduction and induction of virulence activity of native conditional pathobionts^16^. Many clinical reports have demonstrated that gut microbiota dysbiosis can directly cause inflammatory bowel disease and infectious colitis. Dysbiosis is also considered to be one of the most important factors related to other diseases including obesity, necrotizing entrocolitis, type I and type II diabetes, irritable bowel syndrome, and colon cancer. Indeed, it is well documented that the adverse effects of antibiotics are due to the long-lasting perturbation of the host GIT commensal microbiota^17^.

Probiotics (mainly *Lactobacillus* and *Bifidobacterium*) positively modulate gut microbiota, offer effective improvement for host GIT homeostasis, and have been widely used in human to reduce cholesterol levels, improve lactose intolerance, alleviate diarrhea, and protect against infections associated with enteric pathogens^18^. Although probiotic strains are naturally commensal bacteria and generally recognized as safe (GRAS), there have been some safety risks associated with the use of specific strains. For example, *Lactobacillus* may cause infections in immunocompromised patients^19^,^20^, and several cases of probiotics bacteremia were reported in patients with immuno-compromise^21^. The predominant focus of the safety risk associated with probiotics consumption lies with the potential for opportunistic infection in immunocompromised patients. However, other risks related to probiotics consumption are rarely documented. The use of probiotics often involves ingestion of vast numbers of live microorganisms^18^,^22^, which maintains a newly established stability of the intestinal microbiota. In this regard, the abrupt suspension of probiotics consumption may have potentials in destroying this homeostasis of host GIT microbiota, especially in immunocompromised groups, who are more prone to gut microbiota dysbiosis under environmental perturbation^23^.

In this study, we describe a novel probiotics-related risk model that is associated with probiotics consumption suspension. We conducted the experiments in tilapia, a species of vertebrate that can be used as an animal model for biomedical and evolutionary studies^24^ due to its strong environmental adaptability, ease of breeding, and low cost. The use of tilapia here mimicked conditions associated with immunocompromised individuals due to the fact that tilapias (as lower vertebrates) harbor inefficient specific immunity^25^. Two *Lactobacillus* strains that can protect tilapia against pathogen infection following continuous administration were investigated in this study. The suspension in the administration of one strain induced higher susceptibility of the host to *A. hydrophila* infection compared with the control group that was administered a non-probiotic diet. The impaired host defense was highly correlated with the ineffective intestinal mucosal binding activity of the strain, resulting in rapid release of the probiotics from the intestinal niche after the cessation of administration, which disordered host gut microbiome and metabolites, and damaged the permeability of the intestinal epithelium, leading to heavier *A. hydrophila* burden in the following challenge and higher tilapia mortality. We propose that weak mucosa-binding probiotics are associated with safety risks in that the rapid release of these probiotics after administration suspension (including cessation) may lead to host gut dysbiosis and amplify the risk of infections by opportunistic pathogens.

## Results

### Probiotics administration suspension induced tilapia susceptibility to the *A. hydrophila* NJ-1 pathogen

**Figure 1:**
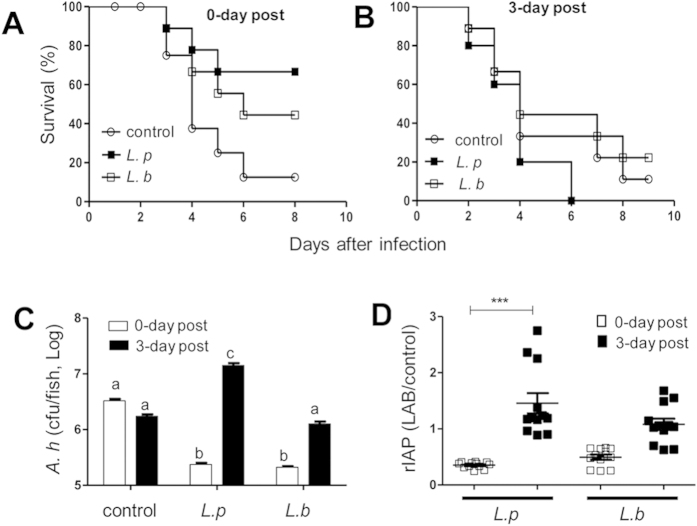
Probiotics feeding suspension increased tilapia mortality and morbidity. Survival curve of tilapia following *A. hydrophila* NJ-1 challenge: tilapias were continuously fed using an experimental diet without (control: empty circle), or with probiotics (*L. p* JCM1149: empty square, *L. b* JCM 1170: filled square). The tilapias were then subjected to a pathogen challenge at (**A**) 0-day post and (**B**) 3-days post probiotics-feeding suspension. (**C**) *A. hydrophila* NJ-1 colonization in tilapias, values with different superscript letters are significantly different (*P* < 0.001). (**D**) Relative IAP (rIAP) activity in tilapias, three asterisks indicate significant difference (*P* < 0.001).

In our previous studies, we demonstrated that both *Lactobacillus plantarum* (*L. p*) JCM1149 and *Lactobacillus brevis* (*L. b*) JCM1170 can protect tilapia against *A. hydrophila* NJ-1 infection following continuous consumption^26^,^27^. In this study, we aim to assess potential risks associated with probiotics administration suspension. Tilapias were continuously fed with an experimental diet supplemented with *L. p* JCM1149 or *L. b* JCM1170 for two weeks. This was followed by a 3-day probiotics administration suspension. The tilapias were subsequently challenged with *A. hydrophila* NJ-1. We confirmed that control specimens that were continuously administered probiotics demonstrated significantly increased host resistance against *A. hydrophila* NJ-1 (Fig. 1A). However, the probiotics-related benefits were completely abrogated following a 3-day probiotics administration suspension (Fig. 1B), i.e., suspension of *L. b* JCM1170 administration produced similar mortality rates as the control group, and suspension of *L. p* JCM1149 resulted in 100% tilapia death when challenged with *A. hydrophila* NJ-1 (Fig. 1B). We further explored the mechanism of protection associated with probiotics treatment and susceptibility during treatment suspension. Both *L. p* JCM1149 and *L. b* JCM1170 exhibited similar inhibitory effects on *A. hydrophila* NJ-1 when an *in vitro* analysis was performed using agar plates (see Supplementary Fig. S1). Additionally, no difference in *A. hydrophila* colonization was observed in tilapia GIT before and after consumption suspension when the experimental diet only was utilized (Fig. 1C, control). However, when specimens exposed to continuous administration of probiotics were evaluated, the burden of *A. hydrophila* were 0.06 fold (*L. b* JCM1170) and 0.07 fold of (*L. p* JCM1149) that of the control group (Fig. 1C, column 5/column 1 and column 3/column 1, respectively), suggesting that probiotics in the intestine can inhibit the binding and/or proliferation of *A. hydrophila*. When specimens exposed to probiotics administration suspension were analyzed, *A. hydrophila* colonization was 7.9 fold (*L. b* JCM1170) and 34.6 fold of (*L. p* JCM1149) that associated with specimens prior to suspension (Fig. 1C, column 6/column 5 and column 4/column 3, respectively). This is consistent with the higher mortality rates shown in Fig. 1B.

IAP can be induced by lipopolysaccharides from Gram negative (G^-^) bacteria, and has been suggested as a biomarker for host response to G^-^ pathogens^28^. We found that there was a linear correlation between relative levels of protection (RLP) of tilapia (over a 30-day period) and IAP units (see Supplementary Fig. S2). Under the probiotics suspension conditions, IAP activity was significantly increased in the group treated with *L. p* JCM1149 but not *L. b* JCM1170 (Fig. 1D). Taken together, the high mortality rate, increased pathogenic *A. hydrophila* burden, and induction of IAP activity suggest that the abrupt suspension of probiotics administration can result in an increased host susceptibility to infection. Since suspension of *L. p* JCM1149 administration caused more serious negative effects compared to *L. b* JCM1170 suspension, we focused on this strain to elucidate the mechanism associated withthis novel risk model.

### *L. p* JCM1149 administration suspension promotes *A. hydrophila* infection efficiency on tilapia intestinal inner surfaces

**Figure 2:**
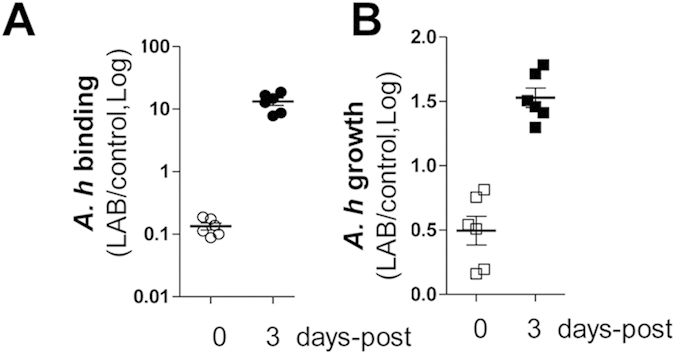
*A. hydrophila* NJ-1 binding (**A**) and proliferation (**B**) on tilapia intestinal tissue after *L.p* JCM1149 feeding suspension. After treatment, tilapia intestines were opened to expose the inner surface. *A. hydrophila* NJ-1 cells were mounted on the inner surface for binding and growth measurement as described in the materials and methods section.

*Ex vivo* intestinal tissue was utilized to assess *A. hydrophila* binding and proliferation in the tilapia GIT. When continuous *L. p*JCM1149 administration was performed, the binding and proliferation of *A. hydrophila* NJ-1 on the intestinal inner surface was 0.1 fold and 0.5 fold of that of the control group, respectively (Fig. 2A,B, first column). When intestinal tissue was collected from tilapia after a 3-day probiotics administration suspension (the suspension followed 14 days of continuous probiotics administration), *A. hydrophila* binding and proliferation increased to 10.0 fold and 1.5 fold of that of the associated control group, respectively (Fig. 2A,B, second column). Interestingly, when dead *L. p* JCM1149 cells were used in the analysis, no significant pathogen binding or growth was observed (see Supplementary Fig. S3). These results indicate that suspension of live *L. p* administration benefits *A. hydrophila* colonization.

### Probiotics administration suspension disrupts tilapia gut homeostasis

**Figure 3:**
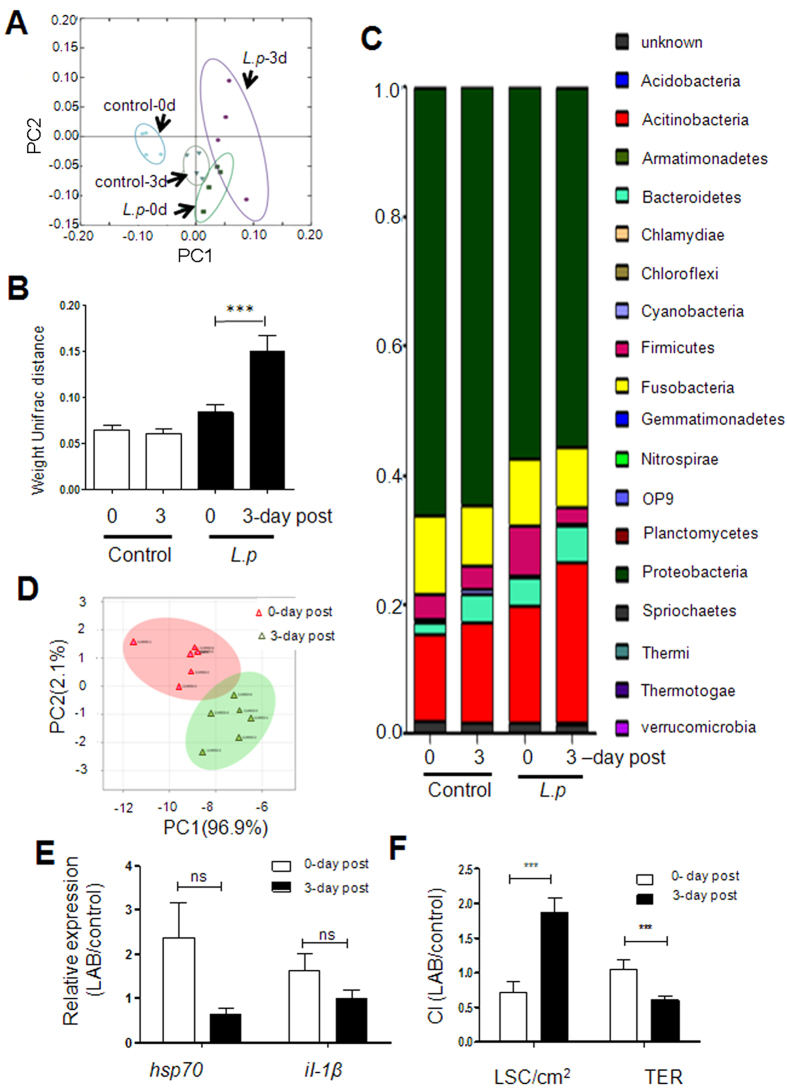
Intestinal homeostasis assessment of tilapias, including Principal coordinate plot (**A**), weighted Unifraction distance (**B**) of observed species distances associated with gut microbiota, staked bar chart (**C**) of the relative abundance of bacterial phylum and OTUs, PCA analysis (**D**) of intestinal metabolites, *q*RT-PCR analysis (**E**) of relative gut *hsp70* and *il-1β* expression, and relative TER and LSC (**F**) of intestinal tissue. Three asterisks indicate significant difference (*P* < 0.001).

The tilapia gut microbiome was analyzed using high throughput 16 S *r*DNA deep sequence technology (V6 region). A total of 1,317,494 unique reads with an average length of 192 bp were generated from 2,311,310 raw reads. A total of 26,142 OTUs were identified from tilapia gut microbiome DNA samples. The OTUs were subsequently analyzed to evaluate relationships among samples based on differences in phylogenetic diversity. Replicates from the control and continuous consumption group were clustered closely when a two-dimensional principal coordinate analysis (PCoA) plot was analyzed. Replicates associated with the probiotics administration suspension treatment were scattered in a disorder fashion (Fig. 3A). We further evaluated these intestinal bacterial communities using weighted UniFrac distances. The analysis showed that dissimilarity in replicates from the *L. p* JCM1149 suspension group was significantly higher compared with the continuous administration group and the control (Fig. 3B). This suggests that the sudden cessation in *L. p* JCM1149 administration disrupted microbiome homeostasis in tilapia GIT. In addition, the alpha-diversity indices, including Shannon, Chao, ACE, and Simpson, were numerically decreased in the probiotics administration suspension group, suggesting an unbalanced microbiota (see Supplementary Fig. S4 and Fig. 3C). The bacterial communities from all groups belonged to 19 phyla, with *Proteobacteria*, *Acitinobacteria*, *Fusobacteria*, *Bacterioidetes*, and *Firmicutes* as the dominant groups. The five phyla constituted over 97.5% of the microbiome. *Proteobacteria* represented the dominant phylum in all the samples analyzed, ranging from 55.6 to 66.3% coverage. When the *L. p* JCM1149 continuous administration group was analyzed, the proportion of *Firmicutes* increased from 0.037% in the control group to 0.079%. When probiotics administration was suspended, this number regressed to 0.027%. Meanwhile, the *Actinobacteria* population was significantly enhanced following *L. p* JCM1149 suspension, with the associated proportion increasing from 0.18% to 0.25%.

We also investigated differences in intestinal metabolite compositions before and after *L. p* JCM1149 suspension. Both principle coordinates analysis (PCA) (Fig. 3D) and partial least squares discriminant analysis (PLS-D) (see Supplementary Fig. S5A ) were used. These analyses sorted the samples into two distinct groups. In total, 15 major metabolites were significantly altered. The concentrations of glucose and 4-aminobutyrate were significantly increased after treatment suspension, while taurine (the main bile acid component) and 12 amino acids (including leucine, valine, alanine, glutamate, phenylalanine, isoleucine, glutamine, lysine, serine, tyrosine, aspartate, and proline) were significantly decreased. Notably, the level of taurine showed the largest difference between the two time points (see Supplementary Fig. S5B).

No difference was observed in the transcription of the intestinal heat shock protein gene *hsp70* and inflammation related gene *il-1β* in response to probiotic suspension (Fig. 3E). This suggested that host immunological responses are not the major reason for increased *A. hydrophila* infection susceptibility.

The “barrier function” of tilapia intestinal epithelium was evaluated by measuring short-circuit currents in intestinal tissue using Ussing Chamber^29^. Significant differences were observed between tilapia intestinal tissues before and after *L. p* JCM1149 suspension (Fig. 3F), indicating that the physical structure of tilapia intestinal epithelium was altered by the abrupt suspension of probiotics administration.

### Antibiotics-induced gut dysbiosis increases tilapia susceptibility to *A. hydrophila* infection

**Figure 4:**
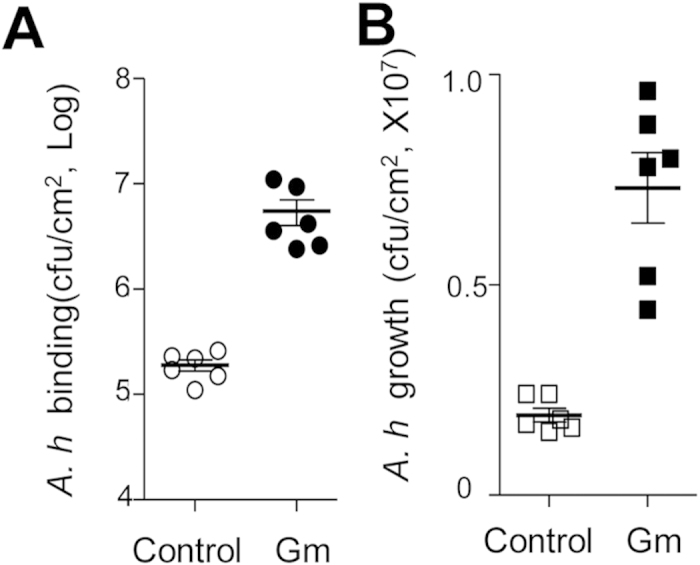
Influence of antibiotics treatment on *A. hydrophila* NJ-1 binding (**A**) and proliferation (**B**) on intestinal surfaces. After antibiotic treatment, tilapia intestines were opened to expose the inner surface, and *A. hydrophila* NJ-1 cells were applied to the inner surface for binding and growth measurement as described in the materials and methods section.

The above results demonstrated that a suspension in the administration of *L. p* JCM1149 induces gut dysbiosis in tilapia. To further validate the relationship between tilapia gut dysbiosis and pathogen susceptibility, antibiotic treatment was performed due to that antibiotic treatment disorders gut homeostasis robustly^30^. The total populations of gut microbiota dropped considerably when tilapia were treated with both kanamycin (Km) and gentamycin (Gm), with the latter showing more optimal bactericidal activity (see Supplementary Fig. S7). As expected, *A. hydrophila* NJ-1 displayed higher binding efficiency (Fig. 4A) and a greater proliferation rate (Fig. 4B) in Gm-treated intestines when compared with the controls. Treatment with Km did not alter the growth of *A. hydrophila* NJ-1 (see Supplementary Fig. S8A), which may be due to the lower bactericidal activity of Km on tilapia gut microbial constituents. Alternatively, this effect may be due to a different spectrum of Km compared with Cm. Even so, Km treatment did increase *A. hydrophila* binding (see Supplementary Fig. S8B).

### Poor gut mucosa binding activity of probiotics was positively related to infection risk following probiotics administration suspension

**Figure 5:**
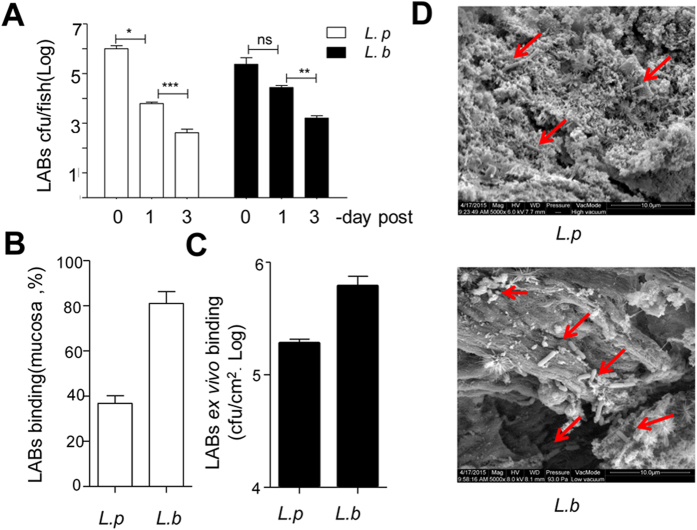
Dynamic kinetics and spatial distribution of probiotics in tilapia intestines. *L. p* JCM1149 and *L. b* JCM1170 populations in intestinal inner surface area (**A**) and intestinal mucosa (**B**) of tilapias. Probiotic binding on tilapia *ex vivo* intestinal tissue was visualized using cell counts (**C**) and SEM (**D**). One, two and three asterisks represent significant differences (*P* < 0.05, *P* < 0.01, and *P* < 0.001, respectively).

In order to determine the mechanism by which probiotics administration suspension induces host susceptibility, we firstly compared the release rate between *L. p* JCM1149 and *L. b*JCM1170 from tilapia GIT as these two strains showed significant differences in host protection against *A. hydrophila* infection after continuous probiotics administration was terminated (Fig. 1B). At 24 hrs post probiotics suspension, the *L. p* JCM1149 population in tilapia GIT decreased dramatically (*P* < 0.05), while no significant difference was observed for *L. b* JCM1170 (Fig. 5A). However, both strains were vigorously released three days after probiotics suspension, with the *L. b* JCM1170 population decreasing from 2.3 × 10^6^ to 1.6 × 10^3^ cfu/tilapia, and the *L. p* JCM1149 population from 1.0 × 10^7^ to 4.1 × 10^2^ cfu/tilapia (Fig. 5A).

The spatial distribution of the bound probiotic cells in tilapia intestinal inner surfacewas quantified after the two-week continuous administration. We observed that 36.8 ± 6.9% *L. p* JCM1149 and 81.0 ± 6.9% *L. b* JCM1170 were located in the tilapia intestinal mucosal zone (Fig. 5B), while others were located in the mucus. This result is consistent with the rapid release of JCM1149 from the tilapia GIT, as the mucus-binding portion of probiotic cells is deemed more loosely attached compared to the mucosal part. The intestinal mucosa-binding efficiency of the two strains was further confirmed using *ex vivo* experiments on intestinal tissue. The probiotics bound to the mucosa were counted on de Man, Rogosa and Sharpe (MRS) agar after serial dilution (Fig. 5C), and were also visualized by scanning electron microscopy (SEM) (Fig. 5D, indicated by red arrows). *L. b* JCM1170 exhibited stronger intestinal mucosa-binding activity than *L. p*JCM1149 on the tilapia intestinal inner surface (Fig 5C,D), which is consistent with the *in vivo* result.

We wondered if this risk model was limited to the *L. p* JCM 1149 strain, or could be extended to additional strains. *Lactobacillus acidophilus* (*L. a*) JCM 1132, *Lactobacillus delbrueckii* subsp. *Bulgaricus* (*L. d*) IMAU20133 and *Lactobacillus fermentum* (*L. f*) IMAU80316, which all belong to *Lactobacillus* but different species, were selected as representative probiotics to perform a comparative analysis. All three strains protected tilapia against *A. hydrophila* NJ-1 infection when the continuous administration condition was applied, as indicated by reduced tilapia IAP activity (see Supplementary Fig. S9A). Conversely, when the 3-day probiotics suspension was applied, IAP activities were significantly induced compared with the control group for each strain (see Supplementary Fig. S9B). Similar to *L. p* JCM1149, the populations of all three strains in tilapia GIT rapidly decreased 24 hrs after probiotics administration was stopped (see Supplementary Fig. S10A). Indeed, most of these strains located in the non-mucosal fraction (see Supplementary Fig. S10B), indicating a common mode of action for risks associated with these probiotic strains.

### Risk model of probiotics administration suspension

**Figure 6:**
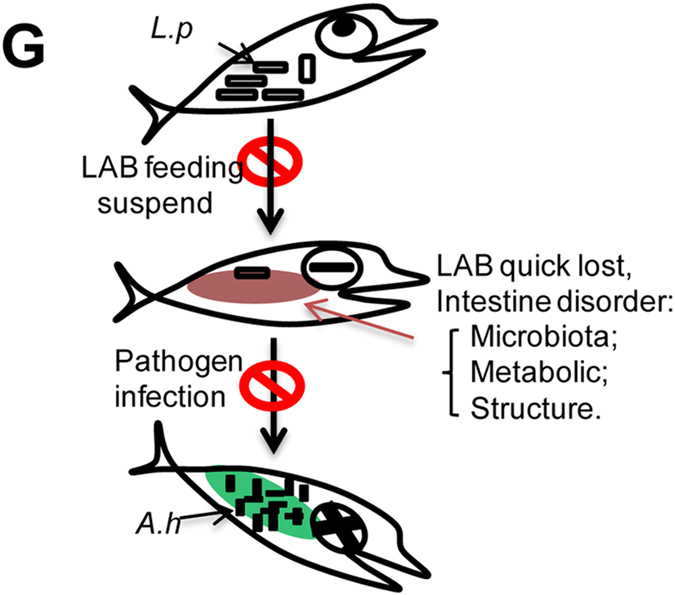
Working model of the risk of probiotics administration suspension. Under continuous feeding conditions, lactic acid bacteria reside in the host intestines and benefit host health. When probiotics administration is suspended, lactic acid bacteria are rapidly released, causing a host intestinal imbalance in the gut microbiota, gut metabolites, and intestinal physical structure. As a result of the gut dysbiosis condition, host pathogens (i.e. *A. hydrophila*) easily infect the host and cause host disease and mortality.

Taken together, we propose the following risk model associated with probiotics administration suspension. During continuous probiotics administration, large quantities of probiotics enter the host GIT, which promote host GIT health and protect against pathogen attack. When probiotics administration is suspended, the probiotic cells are rapidly released, resulting in dysbiosis of the gut microbiome and disruption of the intestinal metabolites and physical function. The resultant GIT environment is more favorable for adherence and proliferation of potential pathobionts, leading to disease development (Fig. 6).

## Discussion

The most important area of safety concern associated with probiotics use is the risk of opportunistic infection, with cases of other safety-related issues rarely reported. In this study, a novel adverse effect associated with probiotics administration was proposed. We observed that tilapia subjected to continuous administration of a probiotic strain (*L. p* JCM1149) followed by 3-days suspension was more susceptible to *A. hydrophila* NJ-1 infection compared with those fed with the control diet throughout the experiment (control group) (Fig. 1B), suggesting the risks of some probiotics in leading to impaired host defense following administration suspension (including cessation). The tilapia model used here simulates the conditions in immunocompromised human groups, for whom the risks proposed here are of most relevance.

The G^-^ strain *A. hydrohpila* is considered a major pathogen in almost all animals, particularly fish. Orally administrated *A. hydrophila* effectively colonizes the intestine, and results in systemic infection in the host^31^. In this study, probiotics were administered to the GIT of tilapia through a feed supplementation regimen. We hypothesized that the change of intestinal environment after probiotics suspension may contribute to the increased tilapia susceptibility to infection. Following PCoA and weighted Unifraction distance analysis, we observed a microbial imbalance (dysbiosis) in the *L. p* JCM1149 group following administration suspension (see Supplementary Fig. 3A–C), which was also suggested by the lower alpha-diversity of the microbiota (see Supplementary Fig. S4). Administration suspension of *L. p* JCM1149 also altered the levels of gut metabolites, generating a different environment compared with that of the continuous treatment group (Fig. 3D, Fig. S4A,B). Taurine, as the main bile acid component, exhibited the largest difference in response to probiotics administration suspension. Bile acids facilitate lipid fat-soluble vitamin absorption in the intestine, and modulate cholesterol metabolism^13^,^32^,^33^. It is well known that the gut microbiota metabolizes bile acids, and subsequently modulates the bile acid profile^34^. Recent research has proven that gut microbes also modulate bile acid synthesis through depression of the nuclear receptor farnesoid X (FXR) pathway^35^, which negatively regulates bile acid synthesis^36^ in the ileum and liver. Therefore, the change in the level of intestinal taurine might be attributed to the gut microbiota alteration after probiotics suspension. Amino acids and glucose are common nutrient components in the intestine and modulation of their metabolism by probiotics or synbiotics has been previously reported in various models^37^,^38^,^39^. The reduction in the level of amino acids suggests that the dysbiosis induced by probiotics consumption suspension may hamper the transportation or absorption function of the intestine. It is well known that amino acids are the main energy sources for the intestine in normal cases^40^. Therefore, the accumulation of glucose and 4-aminobutyrate may be a response effect of the host to the dysbiosis, as both glucose and glutamate (4-aminobutyrate is the intermediate of glutamate catabolism) are important energy compounds in the intestine, which may compensate for the reduction in the levels of amino acids. The dramatic variation of metabolites caused by *L. p* JCM1149 administration suspension further validated that probiotics administration suspension influences gut homeostasis.

Considering the dysbiosis of the gut microbiota and metabolites, we expected that an increase in expression of *hsp70* and *il-1* would be observed following suspension of probiotics administration. Surprisingly, there was no significant difference observed in the expression of *hsp70* and *il-1* following probiotics administration suspension, which indicated that there was no major shift in immune system functioning after probiotics administration suspension. This might be due to the increased level of 4-aminobutyrate, a neurotransmitter with known anti-inflammatory and anti-stress activities^41^, which may have counteracted the effect of gut dysbiosis induced by the suspension of probiotics administration.

Antibiotics treatment is the most robust method to disrupt host intestinal homeostasis mainly by destroying GIT microbial diversity^30^,^42^. We observed that the pathogen *A. hydrophila* NJ-1 demonstrated more optimal binding and proliferation on the intestinal innersurface when tilapias were treated with the antibiotics Gm (Fig. 4). This suggested that gut microbiota dysbiosis is directly related to pathogen invasion in tilapia. Similar phenotypes were observed in our probiotics administration suspension model, which strongly suggested that the unbalanced gut microbiota resulting from probiotics administration suspension is the main cause of increased susceptibility of tilapia (to pathogen infection). These results are consistent with the studies investigating the risks of antibiotics usage in humans and mice^43^,^44^, also indicating the reliability of the tilapia model.

The *L. p* JCM1149 population in the GIT decreased dramatically 24 hrs post suspension, while no significant difference was observed for *L. b* JCM1170. The release rate of the two strains is in agreement with the levels of associated host susceptibility that was induced following the administration suspension. This suggests that the dysbiosis of the gut microbiota may be due to the rapid release of probiotics from the intestines. It has been reported that microbial communities associated with the intestinal inner surface (mucosa and mucus) are distinct from luminal communities and communities associated with feces^45^,^46^. Terminology associated with this subject area is obscure. In much of the literatures, the association of bacteria to the intestinal mucosa actually refers to the intestinal inner surface area including both mucosa and mucus^47^. In this study, the spatial binding pattern of probiotic cells to the intestinal inner surface was evaluated as the distribution of adhered cells associated with mucosa and/or mucus. Results showed that the majority of *L. p* JCM1149 cells were located in the mucus while most of the binding cells of *L. b* JCM1170 located in the mucosal zone. This is consistent with the higher release rate of JCM1149 post administration suspension, which supports the spatial binding pattern of probiotic cells on the intestinal inner surface as the key factor contributing to the risk associated with probiotics administration suspension, i.e., the probiotic strains bound poorly to the mucosal zone are more likely to be rapidly released post suspension, which resulted in dysbiosis of the microbiota and the subsequent disorders, leading to a higher susceptibility of the host to pathogen infection. Notably, *L. p* JCM1149 overall adhered well to the intestinal inner surface area. The numbers of *L. p* JCM1149 cells on the intestinal inner surface of tilapia after 14 days of administration was higher than the numbers of *L. b* JCM1170 cells (Fig. 5A), pointing to a better intestinal binding efficiency. The divergence of this result with the *ex vivo* experiment was probably due to the better overall growth of *L. p* JCM1149 (in comparison with *L. b* JCM1170) in the luminal contents of the intestine, leading to a higher total number of JCM1149 in the intestine surface. Considering the JCM1149 intestinal adherence result, we cannot simply attribute the risk in inducing host susceptibility (to pathogen infection) to poor binding/adherence of probiotics. Probiotic strains with potential risk are most likely those strains demonstrating good overall adherence but undesirable spatial binding patterns, i.e. higher distribution in the mucus zone. Good adherence to the intestinal inner surface (mucosa & mucus), which is considered important for the beneficial action, is one of the criteria for selection of probiotic strains^18^,^47^. Results here suggest that adherence to the intestinal inner surface might represent a “double-edged sword” for probiotic strains that bind in low proportions to the mucosal zone.

We further questioned whether this susceptibility phenotype is exclusive to *L. p* JCM1149, or common to many probiotics. *L. a* JCM 1132, *L. d* IMAU20133 and *L. f* IMAU80316 which all belong to *Lactobacillus* genus but different species, were tested for the susceptibility of tilapia (to pathogen infection) following the same procedures. Unfortunately, all selected strains showed spatial binding patterns similar to *L. p* JCM1149. These strains also showed a similar host susceptibility induction phenotype post administration suspension (see Supplementary Fig. S9), which confirmed that this risk is a general phenomenon associated with some probiotic *Lactobacillus* strains. Further work is required to evaluate the extent of this risk in other genera of probiotics.

The host immune system is the key regulator that maintains the host-microbiota homeostasis. Hosts with compromised immunity are more prone to gut microbial dysbiosis, either spontaneously^48^,^49^ or during environmental perturbations such as changes in host diet^23^. The tilapia model was used in this study to mimic the immunocompromised conditions in humans. The inefficient specific immunity in tilapia^25^ might be an important contributing factor in the gut dysbiosis induced by rapid loss of probiotic strains (as an environmental perturbation), which was supported by the nonsignificant difference in the expression of *hsp70* and *il-1β* following probiotics administration suspension. Speculatively, the observed gut dysbiosis and increased pathogen susceptibility after probiotics administration suspension may also occur in humans with immuno-compromise, which deserves further investigation.

Taken together, we propose a novel risk model for probiotics administration suspension. To our knowledge, this is the first report detailing that the suspension (including cessation) of probiotics administration can increase host susceptibility to infectious disease. It is possible that inactivated probiotics preparations may circumvent this potential risk, but unfortunately some probiotic benefits are directly related to strain viability^50^,^51^. The risk proposed in this study is of particular relevance for immunocompromised patients, or neonatals, where the gut dysbiosis and induced susceptibility to opportunistic pathogens might cause serious problems. Notably, there is currently no direct evidence that this risk exists in mammals and humans, and such a risk model in immunocompromised patients remains theoretical. Nevertheless, the results in our study may provide some insight into risks associated with probiotics consumption, and may guide future research and selection criteria of probiotic strains.

## Methods

### Bacterial strains and growth conditions

Lactic acid strains *L. brevis* JCM1170^27^, *L. plantarum* JCM1149^26^, *L. acidophilus* JCM1132^27^, *L. delbrueckii* subsp. *Bulgaricus* IMAU20133 and *L. fermentum* IMAU80316 (*L. delbrueckii* subsp. *Bulgaricus* IMAU20133 and *L. fermentum* IMAU80316 were gifts from Prof. Zhang Heping, Inner Mongolia Agricultural University, China) were routinely propagated in de Man, Rogosa and Sharpe broth (MRS)^27^ at 37 °C in a static incubator unless otherwise stated. The fish pathogenic strain, *A. hydrophila* NJ-1 (a gift from Dr. Yongjie Liu, Nanjing Agricultural University), was aerobically cultivated in LB broth at 30 °C, with 200 rpm shaking. The cells in stationary phase were harvested through centrifugation at 1500 *g* for 5 min. The cells were subsequently washed three times in phosphate-buffered saline (PBS), and resuspended in PBS at a final concentration of 1.0 × 10^10 ^cfu/ml for *A. hydrophila* NJ-1, and 1.0 × 10^9 ^cfu/ml for lactic acid bacteria, respectively.

### Tilapia husbandry and administration

Hybrid tilapia (*O. niloticus*♀ × *O. aureus* ♂) fingerlings (average body weight, 0.9 g) were purchased from Hainan Tilapia Hatchery (Hainan, China), and acclimatized to laboratory conditions using circulation aquaculture systems (25.2–28.5 °C, flow-through dechlorinated city water at 0.5 liter/min, >6.0 mg O/l oxygen, <0.02 mg N/l ammonium nitrogen, 12 hr: 12 hr light: dark photoperiod) for at least two weeks prior to testing. The tilapias were fed an experimental diet (produced by Tanshan Jiayuan Feed Co., Tanshan China). The experimental diet consisted of 42.0% crude protein and 7.3% crude lipid. The probiotic cells were administered to tilapia in conjunction with the experimental diet at a concentration of 10^8 ^cfu/g as previously described^27^. Both the control and probiotic treatment groups were fed twice a day. All tilapias were fed to apparent satiation per meal.

Procedures involving animals were performed in accordance with Chinese legislation associated with animal experimentation and the studies were approved by the Ethics Committee of the Feed Institute, Chinese Academy of Agricultural Sciences (2012-ZZG-ZF-001).

### *A. hydrophila* challenge

Pathogen challenge experiments were conducted in tanks (length × width × height: 39 × 39 × 38 cm^3^) using the recirculation aquaculture system at the Feed Research Institute of the Chinese Academy of Agricultural Sciences (Beijing, China). *A. hydrophila* strain NJ-1 cells were diluted into rearing water at a final density of 10^8^ cells/ml, and 200 mg of NH_4_Cl was added to enhance the toxicity of the bacterial pathogen. The water and pathogenic bacteria were changed completely every two days. Cumulative mortalities were recorded over a 10-day period.

### Intestine alkaline phosphatase assay

Tilapias were dissected following MS222 (25 mg/l) anesthesia, and the digestive tract of each specimen was aseptically removed in its entirety, following incision with a sterile scalpel. The intestinal tissues were rinsed three times with PBS buffer, homogenized using a glass homogenizer that had been placed in 4 °C ice-cold water (containing 4:1 v/w cold Tris-HCl buffer (0.05 M Tris, 0.1 M NaCl, 0.01 M EDTA, pH 8.0)), and centrifuged at 12,000 *g* for 20 min at 4 °C. The resultant supernatant was preserved for subsequent enzymatic analyses.

Determination of alkaline phosphatase (EC 3.1.3.1) activity was performed using 4-nitro-phenylphosphate as a substrate as previously described^52^. One unit of alkaline phosphatase activity was defined as the amount of enzyme that hydrolyzed 1 nM of substrate in 1 min.

### Gastrointestinal tract microbiome analysis

Tilapia GIT microbiome DNA was extracted from four replicate tilapia gut samples as previously described^53^ with some modifications^54^. PCR amplification of the bacterial 16 S rDNA V6 region was conducted using GIT genomic DNA as the template and primers V6-F (5′-CGCACAAGCGGTGGAGCAT-3′) and V6-R (5′-TCGTTGCGGGACTTAACCCAAC-3′)^55^. Index sequences (8-bp long, barcode) were added to the 5′ end of each primer. The PCRs were performed in a 50 *μ*l final reaction volume with 20 cycles of 94 °C for 30 s, 56 °C for 45 s, 72 °C for 30 s and a final extension 72 °C for 5 min.

PCR products were purified through gel extraction (TiangenGel purification Kit, China) and quantified using Genequant^TM^ pro Nano drop (GE, USA). The amplicons were assessed for DNA concentration and quality, and were subsequently submitted for sequence analysis using an Illumina Miseq platform in the Beijing Computing Center (Beijing, China). Sequence quality were inspected using FastQC software and filtered using the NGS QC Toolkit (Functional Genomics and Bioinformatics Laboratory, National Institute of Plant Genome Research, India) to remove low quality reads. Qualified sequences were greater than 170 bp and contained the primer and barcode sequences. These sequences also lacked any unrecognized sequence stretches. Data were then denoised (qualified sequences were greater than 170 bp and contained the primer and barcode sequences) and analyzed using QIIME^56^, and were concatenated and sorted according to the barcode sequences. Briefly, OTU mapping was performed and sequences displaying a minimum pairwise identity of 97% were used in the analysis. The most abundant sequences identified following OTU mapping were selected for taxonomic classification.

Alpha diversity and beta diversity metrics were calculated on rarefied OTU tables with OIIME to assess sampling depth coverage using observed species, phylogenetic diversity, Cho1, Shannon’s diversity index, and Good’s coverage. Beta diversity metrics among samples were also calculated with OIIME using weighted Unifraction distances^57^. The distance matrixes were demonstrated by two dimensional principal coordinates analysis (PCoA) plots.

### Gastrointestinal tract metabolite analysis

58Tilapias were cultivated with and without the probiotic *L. p* JCM1149 supplement for 14 days. Probiotic administration was subsequently suspended for each of the specimens. At 0 and 3 days post probiotic administration suspension, 36 tilapias were harvested from each group. Intact intestines were immediately sampled using liquid nitrogen. They were subsequently powderized and stocked at −80 °C. Intestines from six individuals were mixed together to prepare a single sample. Six replicates were required for one group, according to the metabolites analysis instruction (Anachro, Wuhan, China).

Intestinal samples were lyophilized and resuspended in Anachro Certified DSS Standard Solution (ACDSS, 4.13 mM). The samples were then subjected to^1^H-NMR analysis (Anachro, Wuhan, China). Briefly, ^1^H NMR spectra were recorded at 298 K using a Bruker AV III 600 MHz NMR spectrometer (BrukerAnalytische GmbH, Rheinstetten, Germany) equipped with an inverse cryoprobe operating at a proton MR frequency of 600.13 MHz. A Noesygppr1d sequence was applied to suppress the residual water signal.

Original data were exported as the resultant free induction decays (FIDs), and signals were phased and baseline-corrected using Chenomx NMR suite v.7.7 (ChenomxInc., Edmonton, Canada). The metabolites were identified by matching spectral signals to 330 metabolites from the Chenomx 600 MHz Library. Metabolite quantification was performed by comparing the integral of a known reference signal (DSS-d_6_) with the signals derived from a library of compounds containing chemical shifts and peak multiplicities for all of the resonances of the compound being analyzed.

The mean-centered and Pareto-scaled NMR data were analyzed with non-supervised principal-component analysis (PCA) (to provide a graphical overview of data) and supervised partial least-squares discriminant analysis (PLS-DA) (to select the potential differential metabolites). The differences in the metabolite concentrations were evaluated using the Non-parametric Mann-Whitney test and the Kruskal-Wallis test (Version 10.0, Statsoft, Inc.). The critical *p*-value was set at 0.05 and variable importance in the projection (VIP) value was set at 1.0.

### Real-time PCR

Tilapia intestinal and liver tissues were harvested for *hsp70* and *il-1β* expression determination, respectively. Total RNA was purified using the TRIzol Reagent RNA kit (Promega, Germany) as described in the manufacturer’s instructions. cDNA was subsequently synthesized using the ReverTra Ace-α-RT-PCR kit (TOYOBO, Shanghai, China). Real-time PCR was performed using SYBR Green Premix EX Taq TM11 (TaKaRa, Beijing, China) and the iQ5multicolor real-time PCR detection system (Bio-Rad, Beijing, China). The oligonucleotides used for *hsp70* and *il-1β* amplification were as follows: *hsp70*: F-5′-TGCCTTTGTCAGACCGTAG-3′ and R-5′- GTGTCCAACGCTGTCATCAC-3′; *il-1β*: F-5′- TGCACTGTCACTGACAGCCAA-3′and R-5′-ATGTTCAGGTGCACTTTGCGG-3′. The genes encoding *β*-actin and 18 S rRNA were chosen as the internal standard, with primers: *β*-actin gene: F-5′-GCTACTCCTTCACCACCACAG-3′ and R-5′-CGTCAGGCAGCTCGTAACTC-3′; 18 S rRNA: F-5′-GGACACGGAAAGGATTGACAG-3′ and R-5′-GTTCGTTATCGGAATTAACCAGAC-3′. The real time PCR reactions utilized the following conditions: 95 °C for 3 min and then 40 cycles of 95 °C for 20 s, 60 °C for 20 s, and 72 °C for 20 s. Dissociation curves were analyzed to assess the melting temperature for each PCR product.

At least three replicates were utilized for each experiment and data were calculated using the 2^−ΔΔCT^ method^59^.

### Ussing Chamber Experiment

Intact tilapia intestines were cut open and stripped from the seromuscular layer in tilapia Ringer’s solution (mM: NaCl, 140; NaHCO_3_, 10; KCl, 4; NaH_2_PO_4_,2; MgSO_4_, 1; CaCl_2_,1; glucose, 5.5; pH 7.8). Tilapia foregut tissues (5 × 5 mm^2^) were mounted on P20038 clamps using the Easy Mount Ussing chamber system (model VCC MC6; Physiologic Instruments, USA) as previously described^60^. After a 20 min equilibration step, transepithelial electrical resistance (TER) and short circuit currents (ISC) were automatically recorded every 5 min over a one-hour period using Acquire and Analyze software (Physiologic Instruments, USA).

### *Ex vivo* intestine assay

To measure *ex vivo* bacterial (probiotics and pathogens) binding and growth in the tilapia GIT, tilapia intestines were collected under sterile conditions. The intestinal specimens were dissected and the digesta were removed to expose the intestinal inner surface.

For the binding assay, 100 *μ*l of bacteria (10^8 ^cfu/ml) were applied to the inner surface of the intestine, and the samples were incubated at 28 °C for 30 min. After incubation, the intestinal tissue was rinsed three times in 1 × PBS buffer. Each rinse was followed by a 10 s vortex step to ensure that the majority of mucosal bacteria were separated from mucus bacteria. Next, the tissues were homogenized prior to performing cell counts on LB plates (for *A. hydrophila* NJ-1) or MRS plates (for *Lactobacillus* sp.).

To assess *A. hydrophila* NJ-1 growth in tilapia intestinal mucosa, intestines were collected and dissected to remove the digest. The samples were subsequently immersed into same amount of sterilized 1 × PBS buffer (1:1, weight/volume). The diluted samples were then vigorously mixed by vortexing for 3 min (Qilinbeier Voatex, Haimen Qilinbeier, China) to facilitate mucosa extraction. An overnight *A. hydrophila* NJ-1 culture (2 *μ*l) was inoculated into 200 *μ*l of the extracted mucosa, and the mixture was subsequently incubated at 37 °C for 12 hr. The number of *A. hydrophila* NJ-1 colonies in the mixture was calculated following serial dilution, plating on LB agar, and incubation.

### Scanning Electron Microscopy (SEM)

For scanning electron microscope (SEM), tilapia intestines were collected and dissected to expose the inner surface to 10^8^ cfu/ml of *L. p* JCM1149 or *L. b* JCM1170 for 1 hr using a 37 °C incubation step. The intestinal samples were then rinsed three times in sterilized 1 × PBS buffer. Intestinal tissues were fixed overnight with 2.5% glutaraldehyde and 2.0% paraformaldehyde in 0.1 M cacodylate buffer, pH 7.4, at 4 °C. After several buffer washes the samples were post fixed in 2.0% osmium tetroxide for 1 h. The samples were subsequently washed once more in buffer and dehydrated in a series of graded ethanol concentrations. Samples were treated several times with hexamethyldisilazane (HMDS) and were then allowed to air-dry prior to mounting and sputter coating with gold/palladium. SEM images were collected using a Jeol JSM-6301 scanning microscope (JEOL) and analyzed with Image J 1.36 (National Institutes of Health, USA).

## Additional Information

**How to cite this article**: Liu, Z. *et al.* Abrupt suspension of probiotics administration may increase host pathogen susceptibility by inducing gut dysbiosis. *Sci. Rep.* **6**, 23214; doi: 10.1038/srep23214 (2016).

## Supplementary Material

Supplementary Information
