# Supplementary material for: Abrupt suspension of probiotics administration may increase host pathogen susceptibility by inducing gut dysbiosis
Source: Sci Rep. 2016 Mar 17;6:23214. doi: 10.1038/srep23214 (PMC4794715; doi:10.1038/srep23214)
Supplement: Supplementary Information [file srep23214-s1.pdf]

# Abrupt suspension of probiotics administration may increase host pathogen susceptibility by inducing gut dysbiosis

## Running title: Suspension of probiotics induces host susceptibility

Zhi Liu[$], Wenshu Liu[$], Chao Ran[$], Jun Hu, Zhigang Zhou*

[1]Key Laboratory for Feed Biotechnology of the Ministry of Agriculture, Feed Research Institute, Chinese Academy of Agricultural Sciences, No. 12 Zhongguancun South St., Beijing 100081,PR China

[$]Equally contributing authors

*Tel.: +86 10 82106073; fax.: +86 10 82106054; Email: zhouzhigang03@caas.cn

**Supplementary Figure**

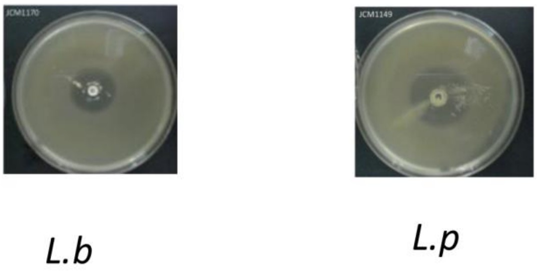


**Supplementary Figure S1.** *In vitro* inhibition of NJ-1 growth by *L. p* JCM1149 and *L.b* JCM1170 using a disc diffusion assay as previouslydescribed[61]. In brief, overnight cultures of *L. p* JCM1149 or *L. b* JCM1170 strains were grown in MRS broth and inoculated as a 5 μl spot on MRS agar and incubated at 37℃ for 24 h prior to initiation of the assay. 100 μl of *A. hydrophila* NJ-1 overnight culture (grown in LB broth) was mixed into 7 ml 0.7% soft LB agar, and was then overlaid on top of the indicator strain. Zones of inhibition around the spots after 24 h incubation at 37℃ were measured and recorded. At least three repeats were performed for each experiment, and representative pictures are shown here.

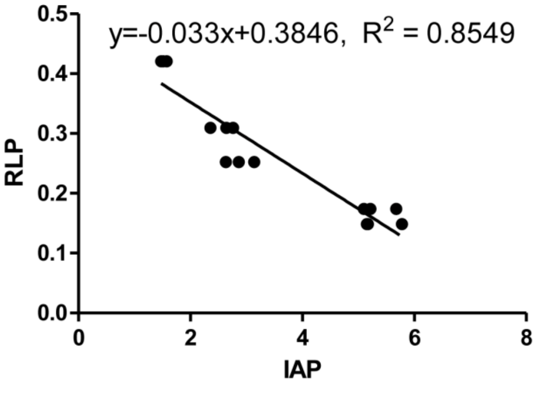


**Supplementary Figure S2.** Linear correlation between IAP activity and relative level of protection (RLP) of tilapias following the *A. hydrophila* NJ-1 challenge. Data were

collected from six independent experiments, in which tilapias were fed with an experimental diet (TanshanJiayuan Feed Co.,Tanshan, China), experimental diet +0.1% Fructooligosaccharide (FOS), experimental diet + *L. b* JCM1170 ($10^8$ cell/g diet), experimental diet + *L. p* JCM1149 ($10^8$ cell/g diet), experimental diet + *L. b* JCM1170 ($10^8$ cell/g diet) + 0.1% FOS, and experimental diet + *L. p* JCM1149 ($10^8$ cell/g diet) + 0.1% FOS. IAP activities were measured 24-hours after the *A. hydrophila* NJ-1 challenge. The RLP value for 10 days was calculated using the following formula; 1 – (mortality/control mortality).

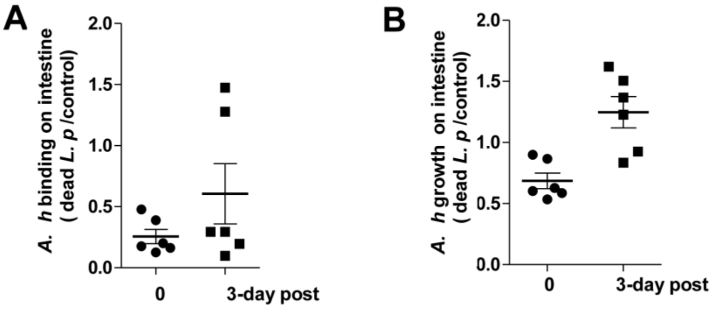


**Supplementary Figure S3.** *A. hydrophila* NJ-1 binding (A) and growth (B) on dead *L. p* JCM1149 treated tilapia intestinal inner surfaces (*ex vivo* model). Binding efficiency and growth were presented as a ratio of the *A. hydrophila* population on the inner surface of the intestines treated with dead *L. p* JCM1149 normalized against the normal feeding control.

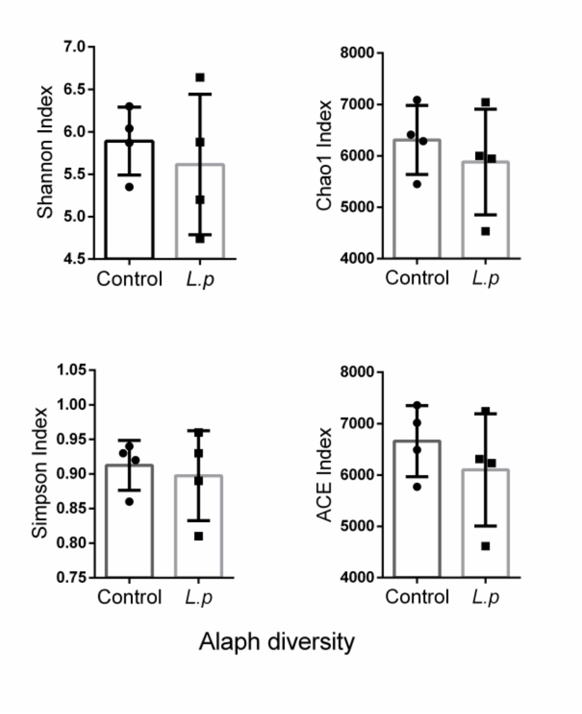


**Supplementary Figure S4.** The alpha-diversity indices of gut microbiota in tilapias with or without *L. p* JCM1149 suspension treatment, including Shannon (A), Chao (B), ACE (C), and Simpson (D).

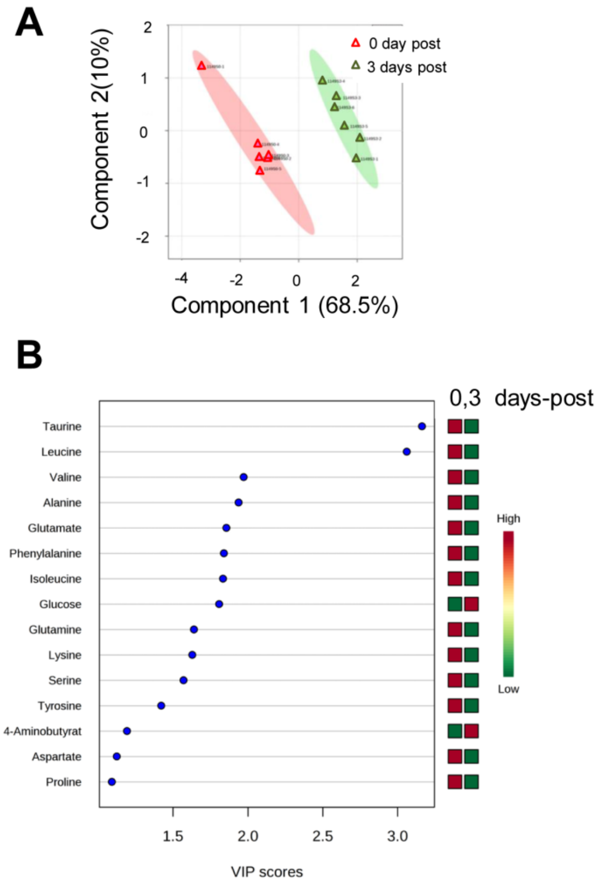


**Supplementary Figure S5.** Intestinal metabolite compositions in tilapias with or without *L. p* JCM1149 suspension treatment. (A) PLS-D assay. (B) Intestinal metabolites VIP score plot.

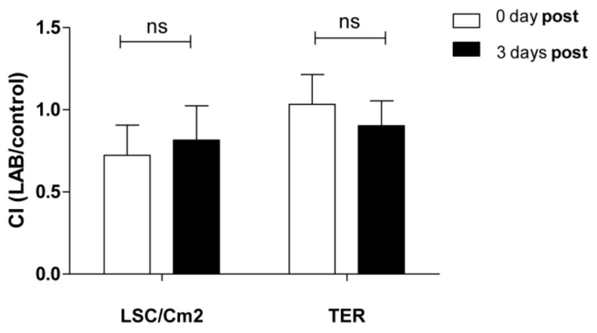


**Supplementary Figure S6.** Relative transepithelial electrical resistance (TER) and short circuit currents (ISC) of intestinal tissue in regular cultivated tilapias. Tilapias

were administrated with an experimental diet, with or without *L. p* JCM1149, for 14 days. They were then subjected to a probiotics administration suspension. Tissue samples were harvested at 0 and 3 days post suspension treatment.

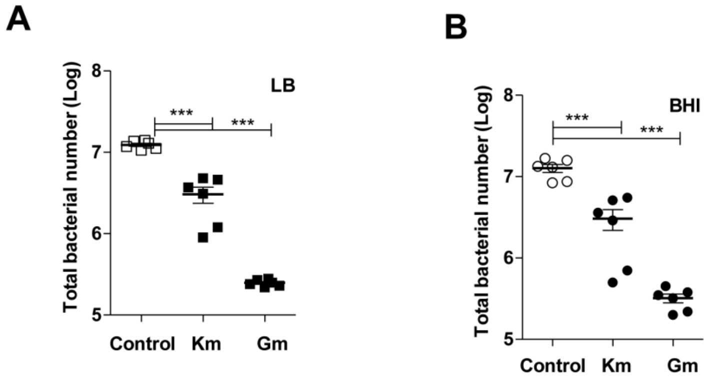


**Supplementary Figure S7.** Antibiotics treatment removed most of the intestinal microbiota measured by cell counting on LB (A) and BHI (B) agar. Three asterisks indicated significant difference (*P*<0.001).

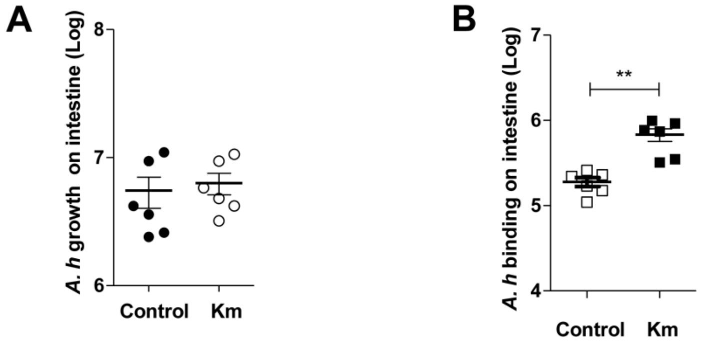


**Supplementary Figure S8.** *A. hydrophila* NJ-1 growth (A) and binding (B) on the intestinal inner surface of Km treated tilapias. Two asterisks indicated significant difference (*P*<0.01).

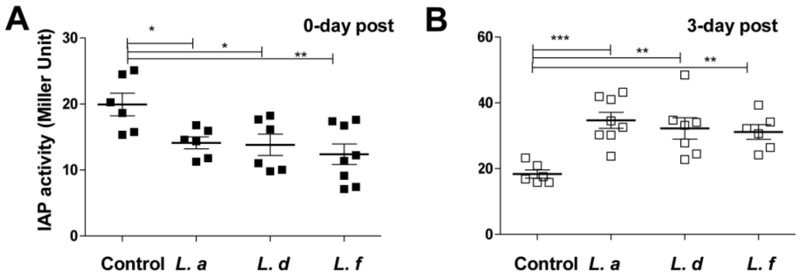


**Supplementary Figure S9.** Assessment of feeding suspension risk of other *L.* spp strains on tilapias by measuring IAP activity at 0 (A) and 3 days (B) post probiotics feeding suspension treatment. *L.a*: *L. acidophilus* JCM 1132. *L.d:L. delbruekii* subsp. *Bulgaricus* IMAU20133. *L. f* : *L. fermentum* IMAU80316.

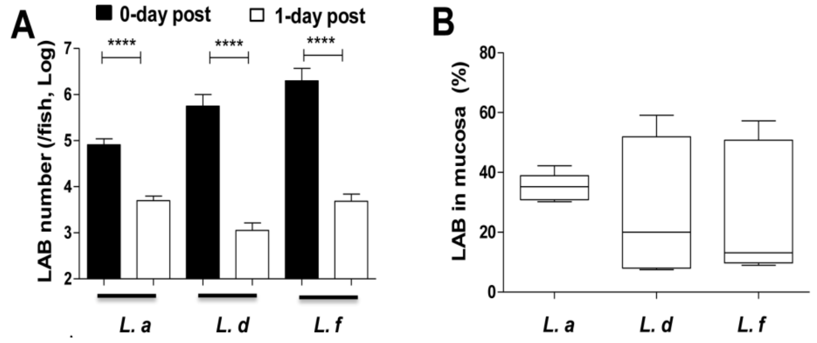


**Supplementary Figure S10.** Probiotics colonization (A) and distribution (B) in tilapia intestinal inner surface.
